# A long-term follow-up of treatment for young children with obesity: a randomized controlled trial

**DOI:** 10.1038/s41366-023-01373-7

**Published:** 2023-10-25

**Authors:** Anna Ek, Markus Brissman, Karin Nordin, Karin Eli, Paulina Nowicka

**Affiliations:** 1 Department of Clinical Science, Intervention and Technology (CLINTEC), Division of Pediatrics, Karolinska Institutet, Stockholm, Sweden; 2 Allied Health Professionals Function, Occupational Therapy & Physiotherapy, Karolinska University Hospital, Stockholm, Sweden; 3 Unit for Biocultural Variation and Obesity, School of Anthropology and Museum Ethnography, University of Oxford, Oxford, UK; 4 Warwick Medical School, University of Warwick, Coventry, UK; 5 Department of Food Studies, Nutrition and Dietetics, Uppsala University, Uppsala, Sweden

**Keywords:** Obesity, Paediatrics

## Abstract

**Background:**

Early childhood obesity interventions supporting parents have the largest effects on child weight status. However, long-term follow-ups are lacking.

**Objective:**

To examine weight status 48 months after obesity treatment initiation for 4- to 6-year-olds.

**Methods:**

177 families were recruited to the More and Less study, a 12-month randomized controlled trial (RCT) conducted in Sweden (2012–2017); 6 children were excluded due to medical diagnoses. Thus, 171 families (non-Swedish origin 59%, university degree 40%) were eligible for this 48-month follow-up with modified intention-to-treat (*n* = 114 had 48-month data, *n* = 34 dropped out, *n* = 23 lost to follow-up). The RCT compared 3 treatment approaches: a 10-week parent support program (1.5 h/w) with follow-up booster sessions (PGB) or without (PGNB), and standard outpatient treatment (ST). Treatment effects on primary outcome (BMI-SDS) and secondary outcomes (BMI, %IOTF25 i.e., the distance, in percent, above the cut-off for overweight) were assessed. Clinically significant reduction of BMI-SDS (≥0.5) was assessed with risk ratio. Sociodemographic factors and attendance were examined by three-way interactions.

**Results:**

After 48 months (mean 50 months, range 38–67 months) mean (95% CI) BMI-SDS was reduced in all groups: PGB −0.45 (−0.18 to −0.73, *p* < 0.001), PGNB −0.34 (−0.13 to −0.55, *p* < 0.001), ST −0.25 (−0.10 to −0.40, *p* < 0.001), no significant difference between groups. A clinically significant reduction of BMI-SDS ≥ 0.5 was obtained in 53.7% of PGB which was twice as likely compared to ST, 33.0%, RR 2.03 (1.27 to 3.27, *p* = 0.003), with no difference to PGNB, 46.6% (*p* = 0.113). %IOTF25 was unchanged from baseline for PGB 4.50 (−1.64 to 10.63), and significantly lower compared to ST 11.92 (8.40 to 15.44) (*p* = 0.043). Sociodemographics or attendance had no effect.

**Conclusion:**

The intensive parent-support early childhood obesity intervention led to better weight status outcomes over time, though BMI-SDS alone did not reflect this. Further research should investigate how to assess weight changes in growing children.

**Clinical trial registration:**

Clinicaltrials.gov, NCT01792531.

## Introduction

Obesity among young children is at unprecedented high levels: in the US, 15% of children under 5 years of age have obesity [1] and in Europe the prevalence of obesity ranges from 3.5% to 7.3% in children age 2–6 years [2]. While most obesity-related comorbidities first appear in young adulthood [3, 4], the trajectory of obesity begins in early childhood [5] and obesity is associated with severe negative effects on children’s physical [6–8] and mental health [9–11]. The strongest predictor for treatment effectiveness is age at initiation [12]; however, few programs are designed for families of young children with obesity [13–16]. Intensive combined lifestyle treatment, starting at the age of 2 years, has been recently proposed by the American Academy of Pediatrics [17]. Only two parent-based treatment programs have been developed for young children (≤6 years old) with obesity and evaluated in randomized controlled trials (RCTs): the Learning about Activity and Understanding Nutrition for Child Health (LAUNCH) program in the U.S. [13, 18] and the More and Less (ML) program in Sweden [14, 19]. Compared to standard treatment, the LAUNCH program led to a more beneficial improvement in the children’s weight status up to 18 months post-baseline, although treatment effects decreased over time [18]. Similarly, the ML program, developed by our team, demonstrated effectiveness in reduction of Body Mass Index standard deviation score (BMI-SDS) and a higher probability of reaching a clinically significant reduction of ≥0.25 and ≥0.5 of BMI-SDS in intervention compared to standard treatment 12 months post-baseline [19].

The purpose of this study was to follow up the diverse population of children who took part in the ML trial, to examine their weight status after 48 months; this allows us to evaluate the long-term effectiveness of a 12-month intensive childhood obesity treatment program. The present study provides a rare opportunity to examine these long-term effects, as only one previous childhood obesity RCT, all age ranges included, has reported results beyond 48 months post-treatment initiation [20].

## Methods

### Design

The ML study was a 12-month open-label, non-blinded, childhood obesity RCT assessing the effects of a 10-week parent support program, with and without telephone-based booster sessions, compared to standard treatment [14, 19]. The study was conducted in Stockholm, Sweden, between March 2012 and October 2017. The study design has been described previously [14, 19]. After the 12-month study, all children were referred to standard care for obesity within the Stockholm Region. The present study reports on the effects on weight status the children experienced 48 months post-baseline, controlling for the treatment the children received after the ML study ended. The trial was approved by the ethics committee in Stockholm (dnr: 2011/1329–31/4) with amendments (dnr: 2012/2005-32, 2013/486-32 and 2016/80-32, for the 48-month follow-up).

### Participants

Families enrolled in the ML study were recruited from 68 primary healthcare centers in the Stockholm region, and some self-referred through advertisements in local newspapers and bulletin boards. Families were eligible for participation if the child was 4-6 years old, had obesity according to the International Obesity Task Force (IOTF) definition [21], and was healthy with no other diagnoses that could affect weight and height development. At least one parent had to understand Swedish well enough to complete questionnaires and participate in parent group sessions delivered in Swedish. Three years after the ML study ended, i.e., 48 months after initiation, families who had participated were invited to the 48-month follow-up visit by letter and phone. After receiving both written and verbal information, those who agreed to participate in the follow-up signed a consent form and were scheduled for measurements.

### Randomization

Families were randomized in a 1:1:2 scheme to the parent support treatment group with (PGB) and without booster sessions (PGNB) and standard treatment (ST), enabling comparison between two groups, parent program (PGB, PGNB) and ST, and between three groups, PGB, PGNB and ST. To reduce possible bias, families and group leaders were blinded to booster/non-booster allocation until the 10-week ML program had finished. Randomization was conducted using an electronic randomization program with permuted blocks by the study statistician, who maintained the randomization list to ensure concealment. A priori power calculation for the 12-month study concluded that 75 children per group (parent program compared to ST) were needed to detect a difference in BMI-SDS of 0.3 (SD 0.5) with 85% power. See Ek et al. [14, 19] for additional information.

### Treatment approaches and settings

#### Intervention

Families in the PGB and PGNB received the ML parent support program. The ML program is based on the Keeping Foster and Kinship Parents Supported and Trained (KEEP) program from Oregon Social Learning Center, US. Details about KEEP [22] and the ML program [14] have been published previously. In short, the manual-based ML program consisted of 10 weekly 90-minute group sessions. Each session was designed to address evidence-based parenting practices that strengthen child-parent communication to facilitate behavior change (e.g., encouragement, positive reinforcement, pre-teaching, effective limit setting and emotion regulation strategies). The parenting practices were discussed and tried out through role plays based on everyday situations that parents find difficult to handle, for example how to cope with a hungry child and how to set up a morning routine without conflicts. Content regarding healthy food choices, appropriate portion sizes and how to balance sedentary behaviors and physical activity was also included in the program to specifically address challenges related to childhood obesity. All parents received a parent manual that covered key information from each session together with handouts. Parents were encouraged to share the manual with other family members and were also encouraged to practice the skills discussed in the groups through home-based assignments. If a parent was absent from a group session, the manual for that session was sent to their home address and a member of the research team called the parent and briefly discussed the session. The ML program was delivered in a healthcare setting by trained group leaders from the research team (2 leaders per parent group). After the 10-week program, PGB received booster phone calls every 4 to 6 weeks over 9 months. Each booster call lasted about 30 min, with a team member providing individually tailored advice and support based on the content of the ML program. During the booster calls the parents were referred to the material in the parent manual.

#### Standard treatment

Families randomized to ST were referred to one of 14 pediatric outpatient clinics for obesity treatment. Both the parent(s) and the child attended treatment based on the action plan for childhood obesity treatment in Stockholm [23], focusing on healthy eating and physical activity. Following the first appointment with a pediatrician, families attended appointments with pediatric nurses, and with dieticians or physiotherapists if needed. The mean number of visits per family in the first year was 5.5 [19]. After each visit (approx. 30 min), the treatment provider sent a description of the treatment the family had received to the research team.

### Measurements and outcomes

At baseline, parents completed sociodemographic questionnaires (see Table 1). Children were measured at baseline, and after 3, 6, 12 and 48 months by the research team or by experienced healthcare professionals in a clinical setting using calibrated instruments. The research team provided a manual to ensure that measurements were taken according to the study protocol. Child weight was measured to the nearest 0.1 kg and height to the nearest 0.1 cm. Waist circumference (WC) was measured to the nearest 0.1 cm using a tape measure between the costa 10 and the iliac crest [24]. All measurements were repeated three times and a mean was calculated. If the family was unable to attend a follow-up appointment, available measurements were obtained from the child’s electronic medical record (Take Care, used by all healthcare providers in Stockholm region).

**Table 1:** Characteristics of the study population at baseline.

				PGB		PGBN		ST
	*n*	mean (sd)	*n*	mean (sd)	*n*	mean (sd)	*n*	mean (sd)
Child								
Age, years	171	5.3 (0.8)	43	5.2 (0.8)	42	5.2 (0.9)	86	5.3 (0.7)
Sex (girl), *n* (%)	171	96 (56.1)	43	19 (44.2)	42	22 (52.4)	86	55 (64.0)
BMI-SDS	171	3.0 (0.6)	43	3.0 (0.6)	42	3.1 (0.7)	86	2.9 (0.6)
BMI, kg/m^2^	171	21.5 (1.9)	43	21.4 (1.5)	42	22.0 (2.3)	86	21.3 (1.8)
%IOTF25	171	123.5 (10.6)	43	122.9 (8.7)	42	126.2 (12.9)	86	122.4 (10.1)
Waist circumference, cm	133	66.8 (5.9)	33	65.2 (4.3)	37	67.8 (6.4)	63	66.9 (6.2)
Weight category, *n* (%)								
Normal weight		-		-		-		-
Overweight		19 (11.1)		4 (9.3)		3 (7.1)		12 (14.0)
Obesity		61 (35.7)		13 (30.2)		13 (31)		35 (40.7)
Severe obesity		91 (53.2)		26 (60.5)		26 (61.9)		39 (45.4)
Mother								
Age, years	136	36.5 (5.4)	30	37.7 (4.7)	34	36.0 (5.5)	72	36.1 (5.7)
BMI, kg/m^2^	138	28.1 (5.8)	31	28.2 (6.1)	35	29.1 (6.6)	72	27.6 (5.1)
Weight category, *n* (%)								
Normal weight		45 (32.8)		9 (29.0)		13 (37.1)		23 (32.4)
Overweight		47 (34.3)		11 (35.5)		9 (25.7)		27 (38.0)
Obesity		25 (18.2)		7 (22.6)		4 (11.4)		14 (19.7)
Severe obesity		20 (14.6)		4 (12.9)		9 (25.7)		7 (9.9)
Migrant origin, *n* (%)	142	86 (60.6)	32	20 (62.5)	36	20 (55.6)	74	46 (62.1)
University degree, *n* (%)	140	57 (41.0)	32	13 (40.6)	35	15 (42.9)	77	29 (39.7)
Income level (SEK per mo), *n* (%)								
<10,000		26 (19.4)		4 (13.8)		7 (21.2)		15 (20.8)
10,000 < 20,000		57 (42.5)		11 (37.9)		12 (36.4)		34 (47.2)
20,000 < 30,000		39 (29.1)		11 (37.9)		9 (27.3)		19 (26.4)
30,000 < 40,000		10 (7.5)		3 (10.3)		4 (12.1)		3 (4.2)
40,000 < 50,000		1 (0.8)		-		-		1 (1.4)
>50,000		1 (0.8)		-		1 (3.0)		-
Father								
Age, years	121	39.5 (7.0)	26	42.7 (7.8)	30	38.2 (7.1)	65	38.8 (6.3)
BMI, kg/m^2^	123	29.5 (4.4)	30	29.0 (4.3)	30	30.2 (4.5)	63	29.4 (4.5)
Weight category, *n* (%)								
Normal weight		14 (11.4)		5 (16.7)		1 (3.3)		8 (12.7)
Overweight		63 (51.2)		17 (56.7)		16 (53.3)		30 (47.6)
Obesity		30 (24.4)		4 (13.3)		7 (23.3)		19 (30.2)
Severe obesity		16 (13.0)		4 (13.3)		6 (20.0)		6 (9.5)
Migrant origin, *n* (%)	127	73 (57.5)	30	17 (56.7)	32	20 (62.5)	65	36 (55.4)
University degree, *n* (%)	125	48 (38.4)	29	11 (37.9)	31	12 (38.7)	65	25 (38.5)
Income level (SEK per mo), *n* (%)								
<10,000		14 (11.7)		3 (11.1)		4 (14.3)		7 (10.8)
10,000 < 20,000		28 (23.3)		7 (25.9)		5 (17.9)		16 (24.6)
20,000 < 30,000		56 (46.7)		11 (40.7)		13 (46.4)		32 (49.2)
30,000 < 40,000		15 (12.5)		3 (11.1)		4 (14.3)		8 (12.3)
40,000 < 50,000		3 (2.5)		1 (3.7)		1 (3.6)		1 (1.5)
>50,000		4 (3.3%)		2 (7.4)		1 (3.6)		1 (1.5)

*BMI* body mass index, *BMI-SDS* body mass standard deviation score defined by International Obesity Task Force, *%IOTF25* % above overweight cut-off defined by International Obesity Task Force, *PGB* parent support program with booster, *PGNB* parent support program without booster, *ST* standard treatment

SEK to US dollar 1 SEK = 0.096 USD (2023-02-24).

The primary outcome of the ML study was mean difference in BMI-SDS between the parent program and ST. BMI was calculated as kg/m^2^ and BMI-SDS was derived from age- and sex-specific reference data [21]. Secondary outcomes were BMI, WC, and cut-offs for a clinically significant reduction of ≥0.25 and ≥0.5 of BMI-SDS, both of which are associated with improvements in metabolic profile in children and adolescents [3, 25]. In addition to these outcomes, we used %IOTF25, a metric similar to %95CDC [26] which has been suggested as an alternative and more appropriate measure than BMI-SDS for long-term follow-up of children with obesity [27–29]. %IOTF25 was calculated as child’s BMI divided by IOTF BMI cut-off for overweight in children (adjusted for sex and age) times 100. A score of 100 equals the cut-off for overweight [27]. Attendance of clinical appointments, specifically for obesity treatment, was derived from questionnaires and medical records. One clinical visit was counted as 30 min, which is an approximation of a standard visit length.

### Statistical analysis

Modified intention-to-treat analysis with linear mixed model compared the effects of treatment on the primary outcome of BMI-SDS, secondary outcomes BMI and WC, and post-hoc outcome %IOTF25 from baseline to 48 months. Data were approximately normally distributed as assessed by histograms. Based on marked differences found between PGB and PGNB at 12 months [19], we chose to compare 3 groups. The main model included group (PGB, PGNB, ST), time (as a continuous variable in months to adjust for variance in follow-up time) and the interaction group*time. Random slope and random intercept were used. We considered time as a categorical factor (i.e., baseline, 3, 6, 9, 12 and 48 months), thus not assuming a linear trend, fitting a model similar to the main model. Both models yielded near identical results (the linear models are presented in Supplemental Fig. 1A–D). Mean differences with 95% confidence interval (95% CI) are reported. Sensitivity analyses were performed by including attendance as well as sociodemographic factors in three-way interactions as well as comparing mode of follow-up assessor (research group vs. medical records). Poisson regression was used to calculate and compare risk ratios (RR) between groups based on the predetermined thresholds of ≥0.25 or ≥0.50 BMI-SDS. RR with 95% CI are reported. The mean of treatment intensity was compared between groups with one-way ANOVA with Bonferroni adjustment. Missing data were considered missing at random and multiple imputation with chained equations was used (m [number of imputations] = 40) [30]. The primary and secondary outcomes and covariates were imputed for all time points (i.e., baseline, 3, 6, 12 and 48 months). All statistical tests were considered two-tailed and p-values below 0.05 were considered significant unless specified. Stata v.15.1 (Stata Corp, College Station, TX) was used for our primary analyses, and SPSS Statistics v.25 was used for descriptive analyses (IBM SPSS Statistics, IBM Corporation).

## Results

**Fig. 1:**
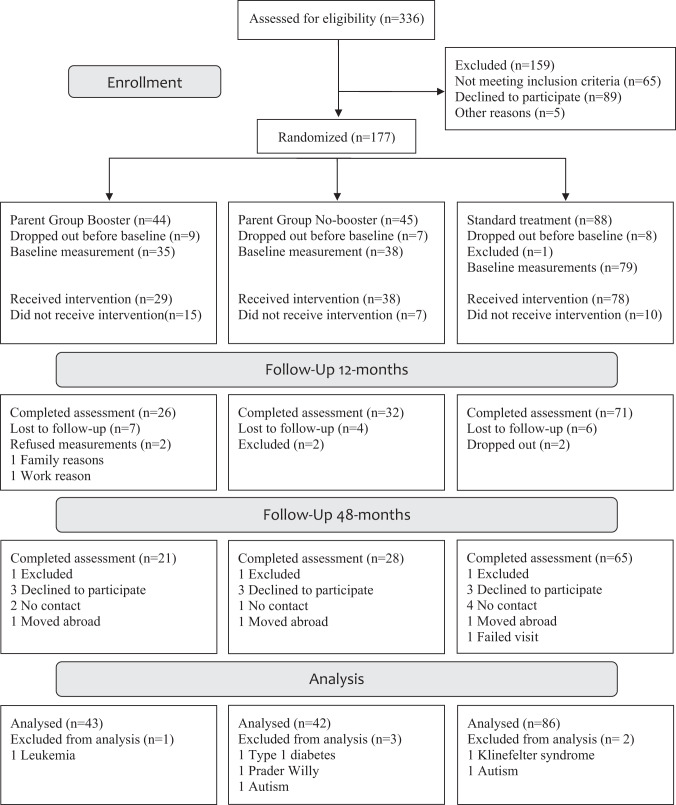
Study participant flow chart.

From the original sample of 177 children, 6 children were excluded due to developing medical diagnoses incompatible with inclusion criteria. Thus, 171 qualified for the modified intention-to-treat-analysis, 19% (*n* = 34) had dropped out between enrollment and 48 months (e.g., before baseline, refused to participate), 13% (*n* = 23) were lost to follow-up (e.g., no contact, moved abroad). At 48 months, 64% (*n* = 114) of the children had measurements; of those, 54% (*n* = 61) were measured by the research team and 46% (*n* = 53) were measured by healthcare professionals, with data collected from medical records. For the 48-month follow-up, measures were collected at mean 50 months, range 38–67 months. See flowchart, Fig. 1. No adverse events were reported.

**Fig. 2:**
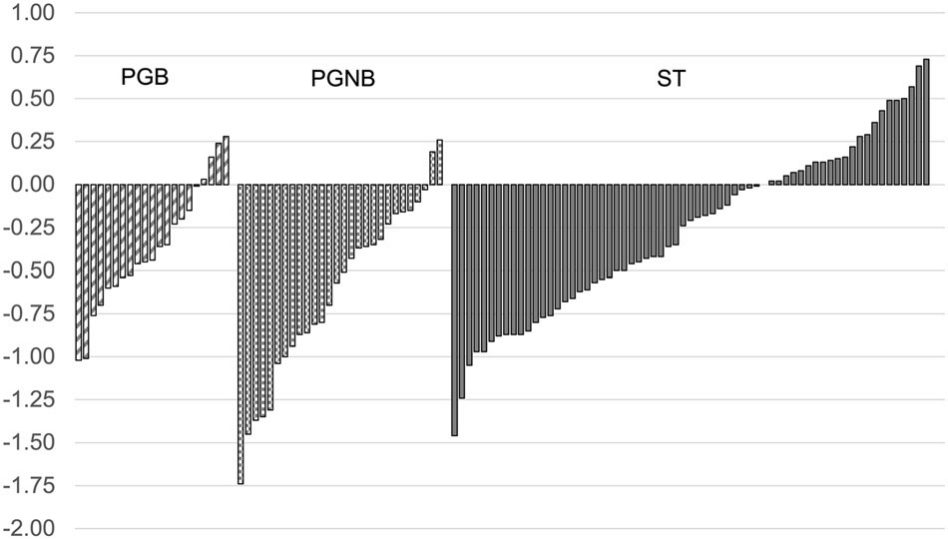
Graphical presentation of individual change in BMI-SDS from baseline to 48 months.

Baseline characteristics are presented in Table 1. At baseline, the mean (SD) age was 5.3 (0.8) years, BMI-SDS was 3.0 (0.6), BMI was 21.5 (1.9) kg/m^2^ and %IOTF25 was 123.5 (10.6). At the 48-month follow-up, the mean (SD) age was 9.5 (0.8) years, BMI-SDS was 2.6 (0.6), BMI was 25.6 (3.8) and %IOTF25 was 132.9 (19.2). Figure 2 illustrates the individual change in BMI-SDS from baseline to 48 months and Supplementary Table 1 presents the sample’s characteristics at 48 months.

### Attendance of clinical visits between 12 and 48 months

Of the 114 children with BMI data (i.e., without imputation), between 12–48 months after treatment initiation, 62% (*n* = 71) attended at least one clinical appointment for obesity treatment. During the 12–48 months period, the mean (SD) number of hours in obesity treatment was 2.2 (2.4) h (range 0–12 h) with no significant differences between groups (Supplementary Table 2).

### Change in weight status (baseline to 48 months)

**Fig. 3:**
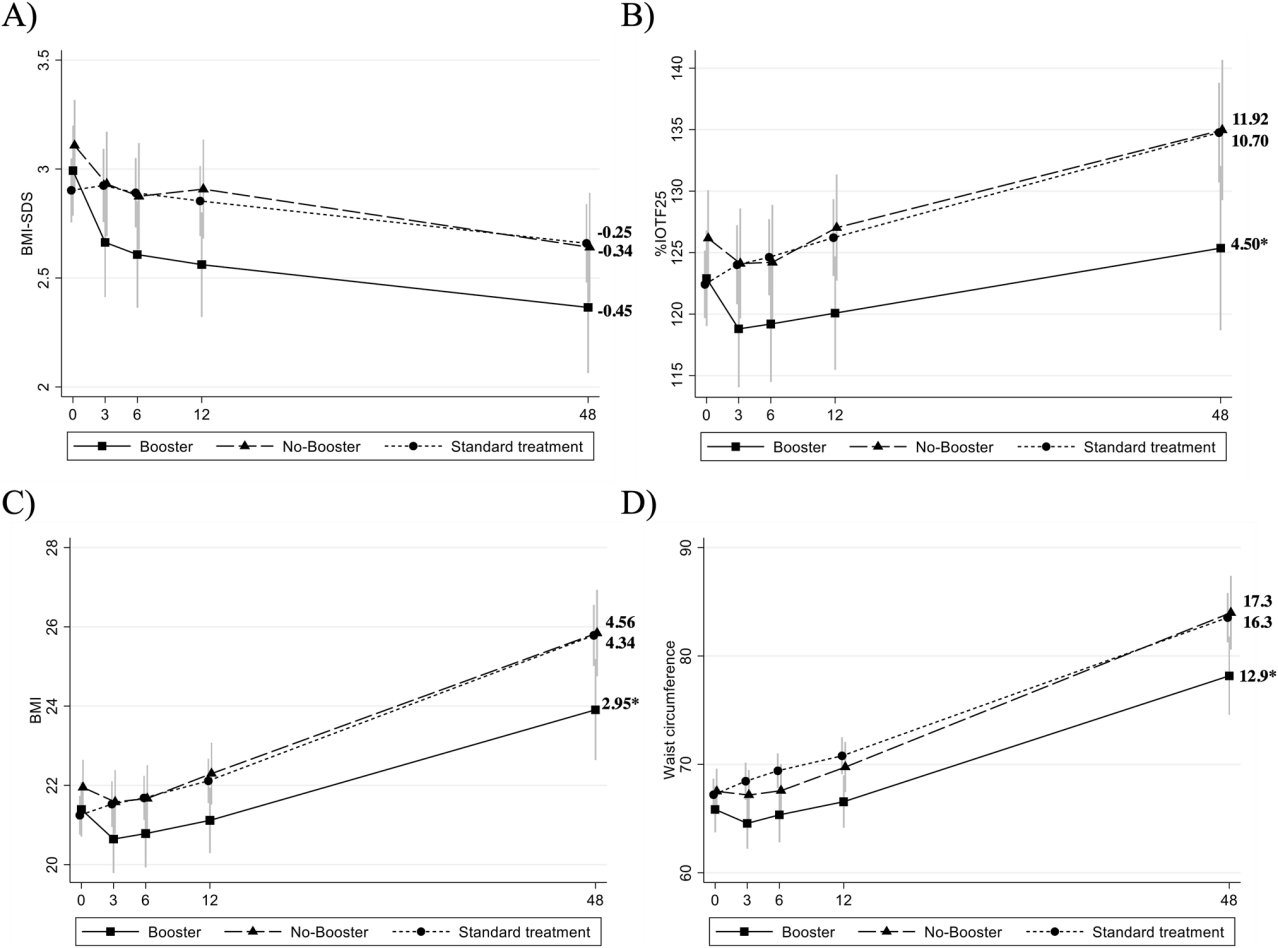
Graphical presentation of change over time from baseline to 48 months (from linear mixed model, with time considered as a factor) for parent support program with booster (Booster), parent support program without booster (No-Booster) and standard treatment. **A** primary outcome BMI-SDS. **B** Secondary post-hoc outcome %IOTF25. **C** BMI. **D** Waist circumference. *P < 0.05 (group difference, ST as a reference in **A**, **B**, **C**, PGNB as a reference in **D**).

In all groups, BMI-SDS, mean (95% CI), decreased over time (baseline to 48 months), for PGB −0.45 (−0.73 to −0.18), PGNB −0.34 (−0.55 to −0.13) and ST −0.25 (−0.40 to −0.10), Fig. 3A. No difference was found between groups (*p* > 0.05), Table 2 and Supplementary Table 3.

**Table 2:** Effect of treatment group by time on primary (BMI-SDS) and secondary (%IOTF25, BMI, WC) outcomes for PGB, PGNB and ST.

	Intercept (SE)^a^	PGB by Time^b^ (95% CI)	P^c^	PGNB by time^d^ (95% CI)	P^c^	Time^e^ (95% CI)	P^f^
BMI-SDS	2.901 (0.048)	−0.004 (−0.011 to 0.002)	0.202	−0.002 (−0.007 to 0.003)	0.455	−0.005 (−0.008 to −0.002)	0.001
%IOTF25	122.472 (0.911)	−0.155 (−0.305 to −0.005)	0.043	−0.026 (−0.149 to 0.098)	0.685	0.248 (0.175 to 0.322)	<0.001
BMI	21.089 (0.162)	−0.033 (−0.062 to −0.005)	0.022	−0.005 (−0.029 to 0.019)	0.695	0.095 (0.081 to 0.109)	<0.001
WC	66.144 (0.476)	−0.070 (−0.150 to 0.010)	0.084	0.021 (−0.055 to 0.097)	0.591	0.339 (0.296 to 0.382)	<0.001

^a^Estimated value for ST at baseline.

^b^Interaction between PGB and time (months) with ST as reference.

^c^*P*-value for the difference between the groups (group by time interaction) with ST as reference.

^d^Interaction between PGNB and time (months) with ST as reference.

^e^Coefficient for time (months) for ST.

^f^The *P* value for change from baseline for ST.

The percentage above the overweight cut-off, mean (95% CI) %IOTF25, increased over time for both PGNB 10.70 (5.70 to 15.70) and ST 11.92 (8.40 to 15.44) but not for PGB 4.50 (−1.64 to 10.63). The increase of %IOTF25 in ST was significant compared to PGB (*p* = 0.043), no difference between PGNB and PGB was found (*p* = 0.117), Fig. 3B, Table 2 and Supplementary Table 3.

Over time, for BMI and WC, mean (95% CI) increased for all groups. PGB had an increase of BMI of 2.95 (1.78 to 4.13), PGNB 4.34 (3.37 to 5.29) and ST 4.56 (3.89 to 5.24). PGB had a smaller increase compared to ST (*p* = 0.022) but similar to PGNB (*p* = 0.071), Fig. 3C, Table 2. For WC (cm), PGB had an increase of 12.9 (9.7 to 16.2), PGNB 17.3 (14.2 to 20.3) and ST 16.3 (14.2 to 18.4). PGB had a smaller increase than PGNB (*p* = 0.039) and no difference between PGB and ST was found (*p* = 0.075), Fig. 3D Table 2 and Supplementary Table 3.

### Clinical significance

At 48 months, the probability (95% CI) of a clinically significant ≥0.5 BM-SDS reduction was twice as likely, RR = 2.03 (1.27 to 3.27, *p* = 0.003), in PGB (53.7%) compared to ST (33.0%). PGNB (46.6%) was not different from ST, RR = 1.51 (0.91 to 2.53, *p* = 0.113). A reduction of ≥0.25 BMI-SDS score was more likely for both PGB (69.8%) RR = 1.84 (1.31 to 2.60, *p* < 0.001) and PGNB (62.6%) RR = 1.56 (1.06 to 2.30, *p* = 0.025) compared to ST (46.7%).

### Shift of weight status category

Table 3 describes the observed data (i.e., without imputation) on weight status category at baseline, 12 months and 48 months for PGB, PGNB and ST. In all groups, a shift to an improved weight status was seen. At 48 months, shifting from severe obesity at baseline to normal weight, overweight or obesity occurred in 14% (*n* = 3) of the children in PGB, 15% (*n* = 4) in PGNB and 13% (*n* = 8) in ST. Shifting from obesity to normal or overweight occurred in 19% (*n* = 4) children in PGB, 12% (*n* = 3) in PGNB and 15% (*n* = 9) in ST. No child shifted to a higher weight status category from baseline to 48 months in PGB. However, at 48 months, 8% (*n* = 5) children in ST had shifted from overweight at baseline to obesity, and one child in PGNB and 7% (*n* = 4) in ST had shifted from obesity to severe obesity.

**Table 3:** Weight status category (normal weight, overweight, obesity and severe obesity) in observed complete cases at baseline, 12 and 48 months by treatment group.

	PGB		PGNB		ST	
	Baseline	48 months	Baseline	48 months	Baseline	48 months
Normal weight, *n* (%)	0	0	0	2 (8)	0	0
Overweight, *n* (%)	3 (14)	8 (38)	2 (8)	4 (15)	10 (16)	16 (26)
Obesity, *n* (%)	10 (48)	8 (38)	7 (27)	6 (23)	27 (44)	25 (41)
Severe Obesity, *n* (%)	8 (38)	5 (24)	17 (66)	14 (54)	24 (39)	20 (33)

Weight status category defined according to International Obesity Task Force (IOTF).

*PGB* parent support program with booster, *PGNB* parent support program without booster, *ST* standard treatment.

### Sensitivity analysis

We investigated if variability in attendance had an effect on the overall findings by including number of visits as a covariate to the primary model; however, no significant effect on the results was found (data not shown). Additionally, we separately analysed the timeframe 12 to 48 months; the results were in the same direction as the main model and coefficients can be found in Supplementary Table 4. Socio-demographic factors, included in three-way interaction analysis, had no influence on the results (data not shown). Missing data analysis found that parents who dropped out between 12 and 48 months were slightly older; no other differences were found between complete and missing data (either lost to follow-up or drop-out). Complete case analysis, i.e., without imputation, can be found in Supplementary Table 5. We also conducted a mode of assessor analysis. When comparing measurements collected by the research nurse (*n* = 61) with those obtained from medical records (*n* = 53) at 48 months we found that 81% (*n* = 17) for PGB, 64% (*n* = 18) for PGNB and 40% (*n* = 26) for ST were taken by the research nurse. Families in PGB and PGNB had more frequent contact with the research team prior to the 48-month follow-up, which may explain the difference. However, there was no difference in change in BMI-SDS, BMI, WC or IOTF% from baseline to 48 months between research nurse and medical records within PGB and PGNB (*p* > 0.05). For ST, BMI was mean (95% CI) −1.6 (−3.1 to −0.1, *p* = 0.033) units lower and IOTF% was −8.0 (−15.1 to −0.9, *p* = 0.027) percent lower in participants with measurements taken by a research nurse compared to those with measurements from medical records.

## Discussion

This study is the first to report on the long-term follow-up of a 12-month obesity treatment among a diverse population of preschool-age children. Weight change was analyzed with four different metrics: BMI-SDS, BMI, WC and %IOTF25. We observed that all groups of children, independently of randomization, reduced their weight status as defined by BMI-SDS from baseline to 48 months with no differences between groups. However, when assessing %IOTF25, a variable more suitable for the comparison of child weight status over a longer period, no reduction of weight status was found. Notably, %IOTF25 for the children who participated in the intensive parent support program followed by booster phone calls (PGB) did not change, whereas both the group without booster sessions (PGNB) and the standard treatment group (ST) experienced an increase in %IOTF25. When evaluating a clinically significant reduction of ≥0.5 BMI-SDS, we found this to be twice as likely in PGB compared to ST. Taken together and in line with our previously reported evaluation of the effectiveness of the ML trial after 12 months [14], long term, the intensive parent support program with booster phone calls remained the more effective obesity treatment compared to standard treatment.

Given the debate on which metric is best when evaluating weight status over time [26–29, 31], we chose to present different dependent measures of child weight status, with the aim of increasing the study’s scientific utility and transparency. The primary outcome in this study, BMI-SDS, was chosen a priori and reflects the state of the science in 2011, when the study was designed [14]. However, when assessing weight status over an extended period of time, the picture is more complex. The distribution of BMI-SDS varies to a large degree in younger ages [28, 32], and may explain why differences between parent-group and ST in our secondary outcomes BMI, IOTF25% and WC were significant, while differences in BMI-SDS were not. The conflicting directions of BMI-SDS and the secondary outcomes should be noted. BMI and WC are expected to increase in growing children; however, %IOTF25, i.e., the distance from overweight, also increased, indicating that the increase of BMI was more pronounced than expected in children of this age group. Still, more research is needed to identify the most accurate metric to assess changes in weight in growing children.

Long-term follow-up of preschool-aged children with obesity who have participated in treatment is scarce, and no other RCT has followed young children with obesity for as long as 48 months [16]. In the other RCT for preschool-aged children with obesity, which was of a similar size to ours, by Stark et al. [13, 18], the initial positive results were not maintained after 18 months. Another, smaller (*n* = 29), study [33] reported results after 3 years, showing an overall effect on BMI-SDS for the intervention group of −0.28 (95% CI −0.54 to −0.03) which can be compared to the −0.25 (95% CI −0.40 to −0.10) seen in ST and the −0.45 (95% CI −0.73 to −0.18) seen in PGB after 48 months in our study. Our findings add evidence that obesity treatment should be initiated early, and that more intense long-term support is likely required to shift the trajectory of weight throughout childhood [17].

Evidence shows that intensive treatment for childhood obesity yields the best results, with more than 26 h per year found to reduce excess weight [34]. In our study, the treatment was most intense during the first 12 months, (PGB (14 h), PGNB (9.5 h) and ST (3.5 h)) [19]. After the 12-month trial, few children received obesity treatment (PGB (1.7 h), PGNB (2.3 h) and ST (2.4 h)), in contrast to the American Academy of Pediatrics recommendation of continuous care [17]. Still, despite the low treatment intensity between 12 and 48 months, we observed that PGB maintained its outcomes compared to PGNB and ST. This was likely due to the magnitude of the initial reduction in weight status achieved by the most intensive treatment group – parent support enhanced by booster phone calls – conveying the importance and benefit of early treatment intensity as well as frequent follow-up.

### Strengths and limitations

This is the largest RCT assessing a parent program for preschool-aged children with obesity. The sample is heterogenous, with most families reporting low or median incomes and diverse ethnic origin, which strengthens the generalizability of our results. A unique feature of this study is the long-term follow-up, which provides insight into what happens during and after a structured obesity intervention. Although 45% of the data at 48 months was derived from medical records, sensitivity analysis showed relatively consistent trends in all groups. The use of a single electronic medical chart system in the Stockholm region along with standardized measurement procedures carried out by well-trained medical personnel leaves little reason to question the validity of these data. Missing data occurred in 30% of participants, which is similar to other long-term follow-up studies; however, 13.6% dropouts occurred before treatment start. To handle missing data, we used what is considered the best approach under the assumption of missing at random [30]. The choice to analyze and compare PGB, PGNB and ST reflects the marked difference seen at 12 months; it should, however, be noted that the study was powered for comparing intervention (PGB and PGNB) against control (ST) (Supplementary Table 6). In addition, the analysis beyond 12 months was exploratory and not pre-specified in the trial registration.

### Future studies

We will examine the other secondary outcomes of ML at 48 months, including child eating behavior, metabolic health, blood chemistry and parental feeding practices and mental wellbeing, to better understand the mechanisms leading from treatment to outcomes. Furthermore, the heterogeneity of treatment response, as shown in Fig. 2, will be investigated in detail.

## Conclusion

To strengthen the evidence-base for early childhood obesity treatment, long-term results from RCTs are needed. In this study, the long-term development of weight status 48 months after an obesity treatment intervention was analyzed through four different metrics. In three out of the four, the most intensive treatment – a parent support program with follow-up booster sessions – emerged as significantly more effective than standard treatment. However, no significant differences between the groups were noted when BMI-SDS results were compared. Given the lack of similar RCTs with long-term follow-up using different measures of weight status, we are likely the first to encounter this discrepancy between metrics. More studies are needed to identify the best measure of weight status in growing children.

### Supplementary information

Supplementary Material

## Data Availability

Data will not be made publicly available but shared upon request by qualified researchers in accordance with approval by regulatory body.
