# Supplementary material for: A long-term follow-up of treatment for young children with obesity: a randomized controlled trial
Source: Int J Obes (Lond). 2023 Oct 25;47(11):1152–60. doi: 10.1038/s41366-023-01373-7 (PMC10599998; doi:10.1038/s41366-023-01373-7)

Supplementary Material

Supplementary Table 1 Characteristics of the observed sample at 48-month follow-up.

Supplemental Table 2 Treatment intensity, hours between 12 and 48 months in observed complete cases stratified by treatment group.

Supplemental Table 3 Effect of treatment group by time on primary (BMI-SDS) and secondary (%IOTF25, BMI, WC) outcomes for PGB, PGNB and ST.

Supplemental Table 4 Effect of treatment group by time, 12 to 48 months, on primary (BMI-SDS) and secondary (%IOTF25, BMI, WC) outcomes for PGB, PGNB and ST.

Supplemental Table 5 Complete-case analysis of effect of treatment group by time on primary (BMI-SDS) and secondary (%IOTF25, BMI, WC) outcomes for PGB, PGNB and ST.

Supplemental Table 6 Effect of treatment group by time on primary (BMI-SDS) and secondary (%IOTF25, BMI, WC) outcomes for PG (PGB and PGNB) and ST.

Supplementary Figure 1 A-D Mean difference change over time for primary (BMI-SDS) and secondary (BMI, %IOTF25 and Waist circumference) outcomes from baseline to 48 months for PGB, PGNB and ST. A, BMI-SDS. B, %IOTF25. C, BMI. D, Waist circumference.

Supplemental Table 1 Characteristics of the observed sample at 48-month follow-up.

		ALL		PGB		PGNB		ST
	n	mean (sd)	n	mean (sd)	n	mean (sd)	n	mean (sd)
Child								
Age, years	114	9.5 (0.8)	21	9.4 (0.8)	28	9.5 (1.0)	65	9.5 (0.8)
Sex (girl), n (%)	171	96 (56.1%)	43	19 (44.2%)	42	22 (52.4%)	86	55 (64.0%)
BMI-SDS	114	2.59 (0.59)	21	2.41 (0.55)	28	2.61 (0.72)	65	2.64 (0.54)
BMI, kg/m2	114	25.53 (3.88)	21	23.95 (2.91)	28	25.87 (4.20)	65	25.89 (3.94)
Waist circumferens, cm	62	80.1 (9.9)	17	78.0 (7.9)	18	81.1 (10.6)	27	80.6 (10.5)
%IOTF25	114	132.9 (19.2)	21	125.1 (15.4)	28	134.4 (20.8)	65	134.7 (19.2)
Weight category, n (%)								
Normal weight		3 (2.6%)		-		3 (10.7%)		-
Overweight		29 (25.4%)		8 (38.1%)		4 (14.3%)		17 (26.2%)
Obesity		39 (34.2%)		8 (38.1%)		6 (21.4%)		25 (38.5%)
Severe obesity		43 (37.7%)		5 (23.8%)		15 (53.6%)		23 (35.4%)
Mother								
Age, years	109	40.5 (5.4)	20	42.2 (5.5)	28	40.3 (5.0)	61	40.1 (5.6)
BMI, kg/m2	63	28.45 (5.80)	15	27.66 (6.4)	19	30.34 (5.99)	29	27.62 (5.24)
Weight category, n (%)								
Normal weight		19 (30.2%)		6 (40.0%)		4 (21.1%)		9 (31.0%)
Overweight		22 (34.9%)		4 (26.7%)		4 (21.1%)		14 (48.3%)
Obesity		11 (17.5%)		2 (13.3%)		6 (31.6%)		3 (10.3%)
Severe obesity		11 (17.5%)		3 (20.0%)		5 (26.3%)		3 (10.3%)
Income level (SEK per mo)								
<10 000		5 (7.8%)		-		1 (5.3%)		4 (12.9%)
10 000 < 20 000		16 (25.0%		5 (35.7%)		6 (31.6%)		5 (16.1%)
20 000 < 30 000		28 (43.8%)		5 (35.7%)		7 (36.8%)		16 (51.6%)

30 000 < 40 000		13 (20.3%)		4 (28.6%)		4 (21.1%)		5 (16.1%)
40 000 < 50 000		-		-		-		-
>50 000		2 (3.1%)		-		1 (5.3%)		1 (3.2%)
Father								
Age, years	100	43.3 (6.6)	19	46.5 (7.2)	25	41.5 (6.2)	56	42.9 (6.3)
BMI, kg/m2	52	29.64 (4.61)	15	28.7 (3.3)	14	31.2 (5.0)	23	29.3 (5.0)
Weight category, n (%)								
Normal weight		7 (13.5%)		2 (13.3%)		0		5 (21.7%)
Overweight		25 (48.1%)		9 (60.0%)		7 (50.0%)		9 (39.1%)
Obesity		11 (21.2%)		3 (20.0%)		3 (21.4%)		5 (21.7%)
Severe obesity		9 (17.3%)		1 (6.7%)		4 (28.6%)		4 (17.4%)
Income level (SEK per mo)								
<10 000		3 (6.0%)		-		1 (7.1%)		2 (9.1%)
10 000 < 20 000		7 (14.0%)		3 (21.4%)		1 (7.1%)		3 (13.6%)
20 000 < 30 000		23 (46.0%)		3 (21.4%)		6 (42.9%)		14 (63.6)
30 000 < 40 000		12 (24.0%)		5 (35.7%)		4 (28.6%)		3 (13.6%)
40 000 < 50 000		4 (8.0%)		2 (14.3%)		2 (14.3%)		-
>50 000		1 (2.0%)		1 (7.1%)		-		-

BMI – Body mass index
BMI-SDS – Body mass standard deviation score defined by International Obesity Task Force
%IOTF25 - % above overweight cut-off defined by International Obesity Task Force
PGB - parent support program with booster
PGNB - parent support program without booster
ST – Standard treatment
SEK to USD 1 SEK = 0.095 USD (2023-02-08)

Supplemental Table 2 Treatment intensity, hours between 12 and 48 months in observed complete cases stratified by treatment group.

		12–24 months		24–36 months		36–48 months		12–48 months	
	n	Mean (sd)	p	Mean (sd)	p	Mean (sd)	p	Mean (sd)	p
PGB	21	0.8 (1.5)		0.3 (0.7)		0.6 (1.1)		1.7 (2.9)	
PGNB	25	0.6 (0.6)	0.238	0.9 (1.0)	0.095	0.8 (1.1)	0.674	2.3 (2.1)	0.493
ST	48	1.0 (1.0)		0.7 (1.0)		0.7 (1.0)		2.4 (2.3)	

PGB: Parent support program with booster
PGNB: Parent support program without booster
ST: Standard treatment

Supplemental Table 3 Effect of treatment group by time on primary (BMI-SDS) and secondary (%IOTF25, BMI, WC) outcomes for PGB, PGNB and ST.

	Intercept[a] (SE)	PGB by Time[b] (95%CI)	P[c]	ST by time[d] (95%CI)	P[c]	Time[e] (95%CI)	P[f]
BMI-SDS	2.901 (0.049)	-0.002 (-0.009 to 0.005)	0.523	0.002 (-0.003 to 0.007)	0.455	-0.007 (-0.012 to -0.003)	0.002
%IOTF25	122.472 (0.911)	-0.129 (-0.291 to 0.033)	0.117	0.026 (-0.098 to 0.149)	0.685	0.223 (0.119 to 0.327)	<0.001
BMI	21.089 (0.162)	-0.029 (-0.060 to 0.003)	0.071	0.005 (-0.019 to 0.029)	0.695	0.090 (0.070 to 0.110)	<0.001
WC	66.144 (0.477)	-0.091 (-0.178 to -0.005)	0.039	-0.021 (-0.097 to 0.055)	0.591	0.360 (0.296 to 0.424)	<0.001

[a] Estimated value for PGNB at baseline.
[b] Interaction between PGB and time (months) with PGNB as reference.
[c] The P value for the difference between the groups (group by time interaction) with PGNB as reference.
[d] Interaction between ST and time (months) with PGNB as reference.
[e] Coefficient for time (months) for PGNB.
[f] The P value for change from baseline for PGNB.

Supplemental Table 4 Effect of treatment group by time, 12 to 48 months, on primary (BMI-SDS) and secondary (%IOTF25, BMI, WC) outcomes for PGB, PGNB and ST.

	Intercept (SE)[a]	PGB by Time[b] (95%CI)	P[c]	PGNB by time[d] (95%CI)	P[c]	Time[e] (95%CI)	P[f]
BMI-SDS	2.858 (0.070)	-0.005 (-0.012 to 0.003)	0.205	-0.001 (-0.007 to 0.005)	0.769	-0.004 (-0.008 to -0.001)	<0.001
%IOTF25	122.212 (1.441)	-0.159 (-0.325 to 0.006)	0.059	-0.007 (-0.143 to 0.130)	0.925	0.254 (0.161 to 0.346)	<0.001
BMI	20.739 (0.257)	-0.034 (-0.066 to -0.003)	0.035	-0.001 (-0.027 to 0.025)	0.938	0.103 (0.086 to 0.121)	<0.001
WC	65.099 (0.800)	-0.089 (-0.177 to -0.001)	0.049	0.019 (-0.064 to 0.101)	0.661	0.370 (0.316 to 0.424)	<0.001

[a] Estimated value for ST at 12 months.
[b] Interaction between PGB and time (months) with ST as reference.
[c] P-value for the difference between the groups (group by time interaction) with ST as reference.
[d] Interaction between PGNB and time (months) with ST as reference.
[e] Coefficient for time (months) for ST.
[f] The P value for change from baseline for ST.

Supplemental Table 5 Complete-case analysis of effect of treatment group by time on primary (BMI-SDS) and secondary (%IOTF25, BMI, WC) outcomes for PGB, PGNB and ST.

	Intercept (SE)[a]	PGB by Time[b] (95%CI)	P[c]	PGNB by time[d] (95%CI)	P[c]	Time[e] (95%CI)	P[f]
BMI-SDS	2.912 (0.051)	0.003 (-0.001 to 0.007)	0.176	-0.004 (-0.008 to -0.00008)	0.045	-0.006 (-0.008 to -0.003)	<0.001
%IOTF25	122.679 (0.911)	-0.019 (-0.145 to 0.107)	0.766	-0.075 (-0.190 to 0.040)	0.199	0.228 (0.165 to 0.291)	<0.001
BMI	21.156 (0.159)	-0.010 (-0.035 to 0.016)	0.458	-0.013 (-0.037 to 0.010)	0.259	0.090 (0.077 to 0.103)	<0.001
WC	66.337 (0.512)	0.015 (-0.065 to 0.010)	0.713	0.035 (-0.044 to 0.113)	0.386	0.284 (0.236 to 0.334)	<0.001

[a] Estimated value for ST at baseline.
[b] Interaction between PGB and time (months) with ST as reference.
[c] P-value for the difference between the groups (group by time interaction) with ST as reference.
[d] Interaction between PGNB and time (months) with ST as reference.
[e] Coefficient for time (months) for ST.
[f] The P value for change from baseline for ST.

Supplemental Table 6 Effect of treatment group by time on primary (BMI-SDS) and secondary (%IOTF25, BMI, WC) outcomes for PG (PGB and PGNB) and ST.

	Intercept[a] (SE)	PG by Time[b] (95%CI)	P[c]	Time[d] (95%CI)	P[e]
BMI-SDS	2.893 (0.048)	-0.001 (-0.005 to 0.003)	0.584	-0.006 (-0.008 to -0.003)	<0.001
%IOTF25	122.665 (0.911)	-0.053 (-0.149 to 0.042)	0.275	0.228 (0.165 to 0.291)	<0.001
BMI	21.075 (0.160)	-0.011 (-0.032 to 0.010)	0.321	0.093 (0.079 to 0.107)	<0.001
WC	66.109 (0.458)	-0.010 (-0.072 to 0.051)	0.740	0.338 (0.294 to 0.382)	<0.001

[a] Estimated value for ST at baseline.
[b] Interaction between PG and time (months) with ST as reference.
[c] The P value for the difference between the groups (group by time interaction) with ST as reference.
[d] Coefficient for time (months) for PGB.
[e] The P value for change from baseline for PGB.

Supplementary Figure 1 A-D Mean difference change over time for primary (BMI-SDS) and secondary (%IOTF25, BMI and waist circumference) outcomes from baseline to 48 months for PGB, PGNB and ST. A, BMI-SDS. B, %IOTF25. C, BMI. D, Waist circumference. * P < 0.05 (group difference, ST as a reference in A, B, C, PGNB as a reference in D).

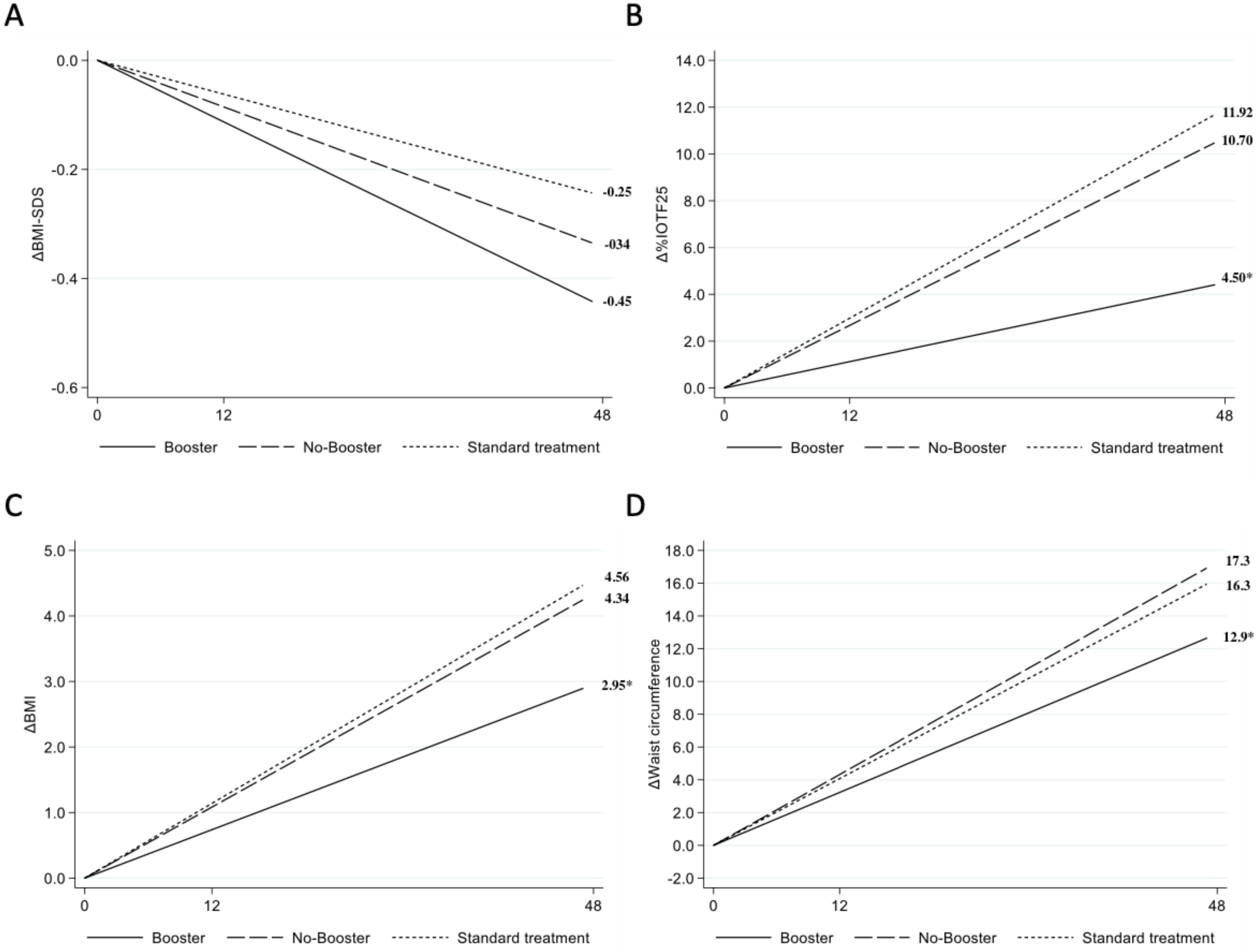

Supplement: Supplementary file 1 — Supplementary Material [file 41366_2023_1373_MOESM1_ESM.pdf]
